# Feasibility of Computed Diffusion Weighted Imaging and Optimization of b-value in Cervical Cancer

**DOI:** 10.2463/mrms.mp.2015-0161

**Published:** 2016-09-16

**Authors:** Yusaku Moribata, Aki Kido, Koji Fujimoto, Yuki Himoto, Yasuhisa Kurata, Fuki Shitano, Kayo Kiguchi, Ikuo Konishi, Kaori Togashi

**Affiliations:** 1 Department of Diagnostic Imaging and Nuclear Medicine, Graduate School of Medicine, Kyoto University, 54 Shogoin Kawahara-cho, Sakyo-ku, Kyoto, Kyoto 606-8507, Japan; 2 Department of Gynecology and Obstetrics, Graduate School of Medicine, Kyoto University, Kyoto, Japan

**Keywords:** cervical cancer, computed diffusion weighted imaging, b-value, magnetic resonance imaging

## Abstract

**Purpose:**

To evaluate the feasibility of computed diffusion weighted imaging (DWI) in cervical cancer and investigate the optimal b-value using computed DWI.

**Methods:**

The present retrospective study involved 85 patients with cervical cancer in the International Federation of Gynecology and Obstetrics (FIGO) stage IB, IIA or IIB. DWI was obtained with b-values of 0, 100, 500 and 1000 s/mm^2^. Computed DWI with b-values of 800, 1000, 1300, 1600 and 2000 s/mm^2^ (cDWI_800_, cDWI_1000_, cDWI_1300_, cDWI_1600_, cDWI_2000_) were generated from all measured DWI (mDWI) data. Qualitatively, computed DWI was evaluated in terms of tumor conspicuity, signal suppression of the fat in the imaged area and total image quality by two radiologists independently with reference to mDWI with b-value of 1000 s/mm^2^. The b-value at which the signal of the endocervical canal was suppressed was recorded. Quantitatively, the signal intensities of tumor, myometrium, endocervical canal, endometrium, and gluteal subcutaneous fat were measured and represented as contrast ratios (CR).

**Results:**

Regarding tumor conspicuity and total image quality, significantly higher scores were obtained at cDWI_1300_ and cDWI_1600_ compared to the others (post-hoc comparison, *P* < 0.001), except for the total image quality between cDWI_1000_ and cDWI_1600_ in one reader. Signal suppression of the fat was the worst at cDWI_2000_. The signal intensity of the endocervical canal was suppressed in 24/27 cases on cDWI_1600_ and in 26/27 cases on cDWI_2000_. The CRs of tumor to myometrium, cervix, and endometrium increased with higher b-values, while the CRs of tumor to fat decreased and were statistically significant (post-hoc comparison, *P* < 0.001).

**Conclusion:**

Computed DWI with the b-values of 1300 and 1600 would be suitable for the evaluation of cervical cancer due to good tumor conspicuity.

## Introduction

The recent promotion of early detection program of cervical cancer has led to an increased detection of cervical cancer at an early stage.^1^ In addition, the aging of pregnant women due to progress in the treatment of infertility has increased the demand for fertility-sparing surgical options such as radical trachelectomy. This procedure involves cervical amputation and excision of parametrial tissue with preservation of the uterine body and the ovaries.^2^ Therefore, the accurate evaluation of tumor extent is critical to make a clinical decision and to select an optimal treatment.

Advances in MRI techniques have improved the resolution and contrast of diffusion weighted imaging (DWI) and DWI is increasingly used in body imaging.^3^ Several studies have reported that DWI could be successfully used for the staging of gynecologic cancer.^4–7^ DWI can also improve diagnostic performances in respect of local invasion in cervical cancer.^8^ One of the major drawbacks of DWI in the evaluation of cervical cancer lies in the high-signal intensity of endocervical canal even on DWI with a b-value of 1000 s/mm^2^. Sometimes this high signal of cervical canal prevents exact assessment of tumor extent in cervical cancer. We thus hypothesized that the high-signal intensity of a normal endocervical canal on DWI may be suppressed using DWI with higher b-values, since high-signal intensity is caused by T_2_ shine-through. If the tumor still remains conspicuous on DWI with high b-value, this may help with a better delineation of the cervical tumor.

Recently, several studies have reported that computed DWI in the body makes it possible to obtain higher b-value images with a good signal-to-noise ratio (SNR) due to the effective suppression of background tissue.^9^ In the matter of prostatic cancer, Ueno et al. have reported that computed DWI with a b-value of 2000 s/mm^2^ appears to be more effective than measured original DWI with a b-value of 1000 s/mm^2^ for the detection of prostatic cancer.^10^ An advantage of computed DWI is that images can be obtained without stress for the patients.

The purpose of the present study was to evaluate the feasibility of computed DWI in cervical cancer and investigate the optimal b-value using computed DWI with b-values of 800, 1000, 1300, 1600 and 2000 s/mm^2^.

## Materials and Methods

### Patients

The present retrospective study was approved by the institutional review board of our institution, and written informed consent was waived. 164 consecutive patients with cervical cancer underwent MR examination at our institution for the assessment of local invasion between January 2010 and July 2014. The inclusion criteria were defined as follows: patients with International Federation of Gynecology and Obstetrics (FIGO) stage IB, IIA and IIB pathologically confirmed by surgery or biopsy. Patients with FIGO stage IA were excluded due to the absence of detectable tumor on MR imaging, while patients with FIGO stage III and IV were excluded because of the difficulty to assess normal surrounding tissue such as endocervical canal, myometrium and endometrium in those patients. Of the 130 patients with stage IB, IIA and IIB, 45 of them were excluded due to the following reasons: strong artifact of DWI (n = 4), undetectable tumor at MRI (n = 27), and tumor with size inferior to 10 mm which was too small to place adequate regions of interest (ROIs) (n = 14). As a result, a total of 85 patients were included in this study. Patients’ ages ranged from 25 to 94 (mean age 53.3 ± 14.9). In total, 45 of the patients were in stage IB, two in stage IIA, and 38 in stage IIB. The pathological diagnosis were squamous cell carcinoma (n = 64), adenocarcinoma (n = 10), adenosquamous carcinoma (n = 5) and small cell carcinoma (n = 3), large cell neuroendocrine carcinoma (n = 2) and carcinosarcoma (n = 1).

### MR imaging technique

MR studies were performed using 3.0T MR units (MAGNETOM Skyra and MAGNETOM Trio; Siemens Healthcare, Erlangen, Germany) and a 1.5T MR unit (MAGNETOM Avanto; Siemens Healthcare, Erlangen, Germany) with a multichannel phased array coil. 3.0T and 1.5T MR units were applied to 72 and 13 patients, respectively. Prior to MR examination, 20 mg of butylscopolamine was administered intramuscularly to reduce bowel peristalsis, unless contraindicated. Fast spin-echo T_2_ weighted images (WI) were acquired in sagittal, axial, and oblique axial planes, and spin-echo T_1_WI were acquired in a sagittal plane.

Sagittal DWI were obtained in the same plane as T_1_WI and T_2_WI using a single-shot echo-planar imaging sequence (TR/TE = 4800/75 ms at 3.0T, TR/TE = 3000/70 ms at 1.5T; field of view (FOV) = 260 mm at 3.0T and 1.5T; slice thickness/intersection gap = 4 mm/1 mm at 3.0T and 1.5T; matrix size: 128 × 128 at 3.0T, 128 × 90 at 1.5T; motion-probing gradients: three directions with b-values of 0, 100, 500, 1000 s/mm^2^ at 3.0T and 1.5T; fat suppression technique: spectral adiabatic inversion recovery (SPAIR) at 3.0T and chemical shift selective (CHESS) at 1.5T; parallel imaging technique: generalized autocalibrating partially parallel acquisitions (GRAPPA) with an acceleration factor of 2 for both 3.0T and 1.5T).

Following acquisition of these images, pre- and post-gadolinium enhanced axial T_1_WI with fat suppression, and contrast-enhanced sagittal spin-echo T_1_WI were acquired.

### Computed DWI

By definition, the signal intensity on DWI exponentially attenuates with b-values based on a measured apparent diffusion coefficient (ADC) value. Therefore, computed DWI is a technique to generate high b-value DWI by extrapolating decaying signal with measured original DWI which is obtained by actual scan.^9^ Based on the following signal model, we calculated ADC value from four measured DWI by nonlinear least square fitting using MATLAB (R2013b, The MathWorks, Natick, MA, USA) and generated computed DWI with b-values of 800, 1000, 1300, 1600 and 2000 s/mm^2^,^11^

$${\text{S}}_{\text{b}}={\text{S}}_{0}\;\text{exp}(-\text{b}\times \text{ADC})$$

where S_b_ and S_0_ corresponded to the signal intensities of a given b-value and that without applying any diffusion gradients, respectively.

### Qualitative analysis

The image quality of DWI was independently scored by two radiologists (Y.K. and Y.H.), who have six and seven years of experience in female pelvic MRI respectively. Computed DWI with b-values of 800, 1000, 1300, 1600, and 2000 s/mm^2^ (cDWI_800_, cDWI_1000_, cDWI_1300_, cDWI_1600_, and cDWI_2000_) were evaluated with reference to measured DWI with a b-value of 1000 s/mm^2^ (mDWI_1000_) and T_2_WI on a representative slice showing cervical tumor. Evaluation points were tumor conspicuity, signal suppression of the fat in the imaged area, and total image quality. Tumor conspicuity and total image quality were scored on a 5-point scale as follows: 1, prominently poorer; 2, poorer; 3, equivalent; 4, better; 5, far better, when compared with mDWI_1000_. Signal suppression of the fat in the imaged area was scored on a 4-point scale as follows: 1, prominently poorer, indicating high-signal intensity of tissues with roughness; 2, poorer; 3, equal; 4, better, indicating well suppressed signals, compared with those of mDWI_1000_.

In addition to the three imaging criteria mentioned above, the signal intensity of endocervical canal was also assessed in 27 out of 85 cases in which the endocervical canal was preserved. The signal was assessed on a representative slice showing the endocervical canal on computed DWI sets. The b-value at which the signal intensity of the endocervical canal was suppressed was recorded by both radiologists.

### Quantitative analysis

The correlation coefficient for the signal intensities of tumor, myometrium, endocervical canal, endometrium, and gluteal subcutaneous fat on mDWI_1000_ and cDWI_1000_ was calculated in order to examine the equivalence between mDWI and cDWI.

Contrast ratios (CRs) of tumor to the myometrium, tumor to endocervical canal, tumor to the endometrium and tumor to the gluteal subcutaneous fat were calculated using the following formula:

$${\text{CR}}_{\text{A}\;\text{to}\;\text{B}\;}=({\text{SI}}_{\text{A}}-{\text{SI}}_{\text{B}})/({\text{SI}}_{\text{A}}+{\text{SI}}_{\text{B}}),$$

where SI_A_ and SI_B_ correspond to the mean values of two different tissues. CRs were compared among the cDWIs obtained with each of the five b-values. The signal intensities of the tumor, myometrium, and gluteal subcutaneous fat were measured in all 85 patient cases. The signal intensities of the endometrium were measured in 70 cases by excluding those with hematometra/pyometra (n = 7), submucosal myoma (n = 3), pregnancy (n = 2), cervical cancer with corpus invasion (n = 1), intrauterine device (n = 1) and thin endometrium (n = 1). The signal intensities of the endocervical canal were measured in the 27 patient cases, which were also included in the qualitative analysis.

The degree of signal decay in the tumor and fat tissue between b-values of 800 and 2000 s/mm^2^ was calculated using the following formula:

$${\text{D}}_{\text{A}}=({\text{SI}}_{\text{A}}(800)-{\text{SI}}_{\text{A}}(2000))/{\text{SI}}_{\text{A}}(800)\times 100(\%),$$

where SI_A_(800) and SI_A_2000 correspond to the mean signal intensities of tumor or fat tissue on cDWI_800_ and cDWI_2000_, respectively.

The signal intensity of each region indicated above was measured by placing freehand ROIs as large as possible. These ROIs were positioned in reference to T_2_WI and mDWI_1000_ by one of the authors (Y.M.), who has 6 years of experience in female pelvic MRI. The same ROIs were applied to all the cDWI sets. The mean value of each ROI was recorded.

### Statistical analysis

Statistical analysis was performed using a commercially available software (MedCalc version 12.7.8.0, MedCalc Software, Belgium). Interobserver agreement on the qualitative analysis was assessed by weighted kappa test with quadratic weighting. The agreement in terms of kappa value was set as follows: <0.20 = poor agreement; 0.21–0.40 = fair agreement; 0.41–0.60 = moderate agreement; 0.61–0.80 = good agreement; 0.81–1.00 = excellent agreement.^12^ The relationship between mDWI_1000_ and cDWI_1000_ was assessed by the scatter plot representation of the signal intensities of all ROIs (tumor, myometrium, endometrium, endocervical canal, and subcutaneous fat), and the correlation coefficient between mDWI_1000_ and cDWI_1000_ was calculated. The scores of qualitative analysis and the CRs for each b-value were compared using Friedman test. A *P*-value of <0.05 was considered statistically significant. If significant differences were found, a post-hoc pairwise comparison was performed using the Wilcoxon signed-rank test with Bonferroni correction, and a *P*-value of <0.005 was considered statistically significant since ten pairs were compared.

## Results

### Qualitative analysis

The scores for tumor conspicuity, signal suppression of the fat in the imaged area, and total image quality are summarized in Table 1. There were good inter-observer agreements between the two readers for tumor conspicuity (κ = 0.68), signal suppression of the fat in the imaged area (κ = 0.75), and total image quality (κ = 0.61). Regarding tumor conspicuity and total image quality, by post-hoc comparison, both readers assigned significantly higher scores on cDWI_1300_ and cDWI_1600_ compared to cDWI_800_, cDWI_1000_ and cDWI_2000_ (*P* <0.001), except for the total image quality between cDWI_1000_ and cDWI_1600_, where there was no significant difference for reader B (*P* = 0.059). There was no significant difference between cDWI_1300_ and cDWI_1600_ on both tumor conspicuity and total image quality. The scores associated with signal suppression of the fat in the imaged area decreased with b-values, and significantly higher scores were obtained on cDWI_800_ and cDWI_1000_ compared to cDWI_1300_, cDWI_1600_, and cDWI_2000_ (*P* <0.001).

The high-signal intensity of the endocervical canal was suppressed in 26 out of 27 cases with increasing b-values. Suppression of the signal intensity of the endocervical canal was observed on either cDWI_1300_ or cDWI_1600_ in most cases (Table 2). There was also a good inter-observer agreement between the two readers (κ = 0.78).

### Quantitative analysis

**Fig 1.**
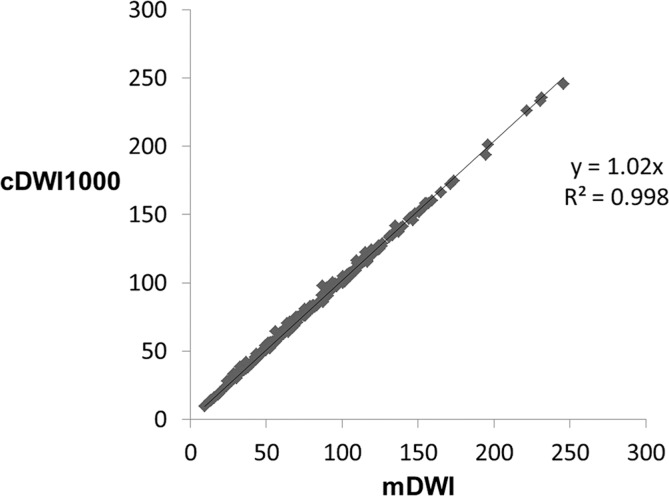
Scatter plots of the signal intensities of tumor, myometrium, endocervical canal, endometrium, and gluteal subcutaneous fat on measured DWI with a b-value of 1000 s/mm^2^ (mDWI_1000_) and computed DWI with a b-value of 1000 s/mm^2^ (cDWI_1000_). The correlation coefficient between mDWI_1000_ and cDWI_1000_ was very high (R^2^ = 0.998), and the linear regression slope was 1.02.

The correlation coefficient between the signal intensities on cDWI_1000_ and mDWI_1000_ was very high (R^2^ = 0.998). The linear regression slope of these two datasets was 1.02 (i.e. cDWI_1000_ = 1.02 × mDWI_1000_), which means that cDWI and mDWI were almost identical (Fig. 1).

**Fig 2.**
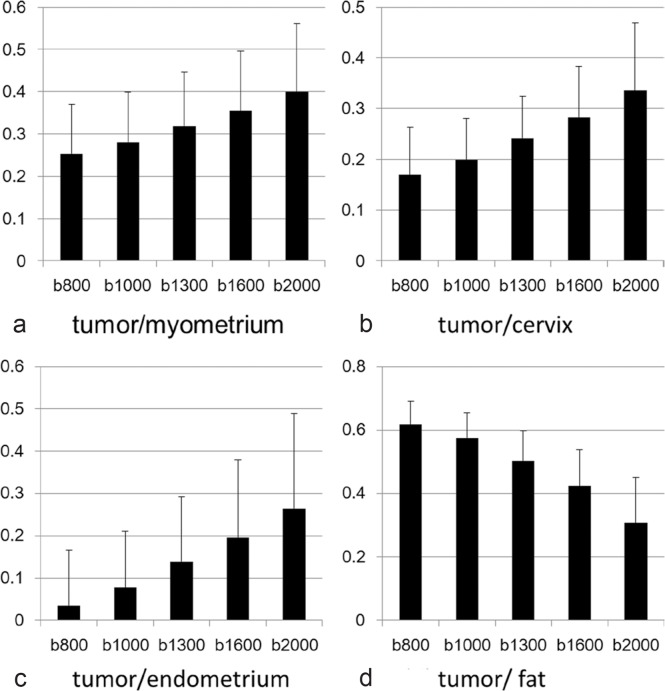
The average and standard deviation of the contrast ratios (CRs) of tumor to myometrium (**a**), tumor to cervix (**b**), tumor to endometrium (**c**) and tumor to fat (**d**) on computed diffusion weighted images are shown. The CR of tumor to myometrium, tumor to cervix and tumor to endometrium increased with increasing b-values, while the CR of tumor to fat decreased with increasing b-values.

The CRs of each tissue combinations are shown in Table 3 and Fig. 2. The CRs of tumor to myometrium, tumor to cervix and tumor to endometrium increased with increasing b-values, while the CR of tumor to fat decreased. Post-hoc comparison revealed that there were significant differences between any pair of b-values in the CRs of each tissue (*P* <0.001 between each pair).

**Fig 3.**
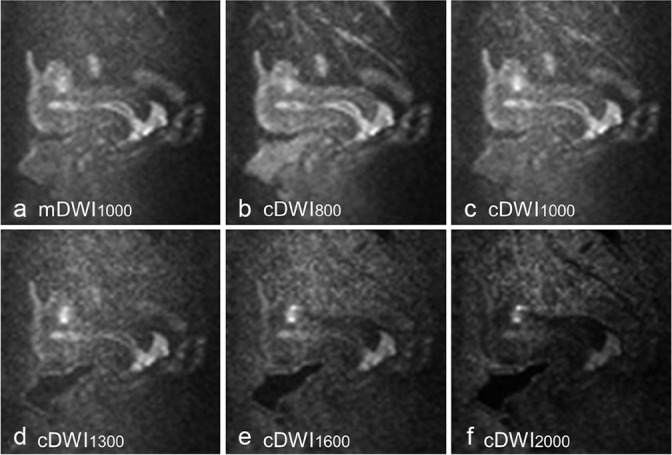
A 32-year-old woman with cervical cancer, squamous cell carcinoma, in FIGO stage IB. (**a**) mDWI_1000_, (**b**) cDWI_800_, (**c**) cDWI_1000_, (**d**) cDWI_1300_, (**e**) cDWI_1600_, and (**f**) cDWI_2000_ are shown. The border between tumor and endocervical canal was obscure on cDWI_800_, cDWI_1000_ and cDWI_1300_, whereas the signal intensity of endocervical canal was suppressed on cDWI_1600_ and cDWI_2000_. Importantly, however, the decreased signal intensity of tumor on cDWI_2000_ resulted in a decline in tumor conspicuity. The contrast between uterus and mesenteric fat is inverted at cDWI_800_ and cDWI_2000_.

**Fig 4.**
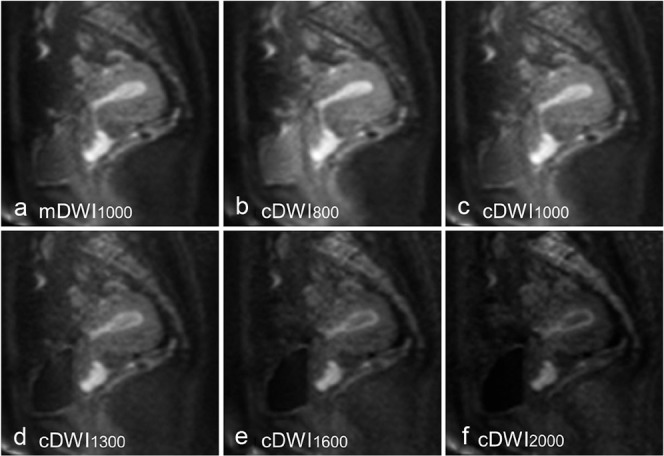
A 31-year-old woman with cervical cancer, adenosquamous carcinoma, in FIGO stage IB. (**a**) mDWI_1000_, (**b**) cDWI_800_, (**c**) cDWI_1000_, (**d**) cDWI_1300_, (**e**) cDWI_1600_, (**f**) cDWI_2000_ are shown. Both the endometrium and tumor showed high signal and appeared as continuous tissues. However, only the tumor was conspicuous on cDWI_1300_ and cDWI_1600_. In addition, the tumor showed lower signal intensity on cDWI_2000_ than that on cDWI_1300_.

Signal decay between cDWI_800_ and cDWI_2000_, for fat (D_fat_: 12%) was lower than that for tumor (D_tumor_: 61%). Representative cases are shown in Figs. 3 and 4.

## Discussion

To the best of our knowledge, no prior study has used computed DWI for the clinical evaluation of cervical cancer. Our results showed that b-values of 1300 and 1600 s/mm^2^ are optimal for the visualization of cervical cancer with respect to the contrast between tumor and its surrounding normal tissue using computed DWI.

The CRs of tumor to fat decreased with increasing b-values, while the CRs of tumor to normal uterine tissues increased. As our results showed that the degree of signal decay of fat was 12%, the signal intensity of fat tissue can be considered relatively constant and independent of variations in b-value. Therefore, a decrease in the CR of tumor to fat on computed DWI with high b-value depends mainly on the signal decay of the tumor. In the case of prostatic cancer, it was reported that periprostatic fat tissue became brighter on computed DWI with increasing b-value because the ADC value of fat tissue was often lower than that of tumor.^13^ Our results on signal decay of tumor with increasing b-values were consistent with these previous results.

The decrease in tumor signal with increasing b-values may also account for the discrepancy of the results between qualitative and quantitative analyses. In the qualitative assessment of tumor conspicuity, both cDWI_1300_ and cDWI_1600_ were assigned high scores. However, the CRs of tumor to normal uterine tissues increased with increasing b-values. This discrepancy may be due to the signal decay of tumor with high b-values, as already mentioned above. On cDWI_2000_, the signal decay of the tumor may have a larger impact on tumor conspicuity than an elevated CR of tumor to myometrium. In addition, since tumor signal decreases with increasing b-values, the increase of CRs may be explained by a greater signal decay of the normal uterine tissues compared to that of tumors.

The endocervical canal usually shows a high signal on mDWI_1000_. In such cases, it was difficult to detect tumor invasion along the endocervical canal on mDWI_1000_. The present study thus suggests that the signal intensity of the endocervical canal was suppressed on computed DWI with high b-values, i.e., 24/27 cases at a b-value of 1600 s/mm^2^. Moreover, if the signal intensity of endocervical canal is not suppressed on cDWI_1300_ or cDWI_1600_, cDWI_2000_ can be available without extra scan and may be helpful. If computed DWI was introduced into clinical settings, this tool may help to determine the indication of fertility-sparing surgery such as trachelectomy. Because the extent of resection is limited in trachelectomy, the indication of patients is strictly limited to those with a tumor size inferior to 2 cm in maximum dimension and those with a distance of at least 1cm between the tumor and the internal os. The high-signal intensity of the endocervical canal on mDWI_1000_ related to T_2_ shine-through effect may be excluded also on ADC map, but it is difficult to assess tumor invasion to cervical stroma on ADC map because cervical stroma also shows low signal intensity on ADC map. DWI makes it easy to detect tumors and has broad utility such as fusion with T_2_WI and maximum intensity projection (MIP) of diffusion weighted whole body imaging with background body signal suppression (DWIBS).^8,14^

To the best of our knowledge, there has been no previous report on the utility of DWI with b-values above 1000 s/mm^2^ for the evaluation of cervical cancer. Our results suggest that computed DWI with b-values of 1300 and 1600 s/mm^2^ would be appropriate to visualize cervical cancer. In the context of prostate cancer, a number of studies have investigated the appropriate b-values to detect tumors with the use of b-values greater than 1000 s/mm^2^ with or without computed DWI.^10,13,15–22^ Though the optimal b-value is still controversial in prostate cancer, higher b-values of 1500 or 2000 s/mm^2^ are supposed to be useful. In contrast, in the context of cervical cancer, our results suggested that a b-value of 2000 s/mm^2^ should not be recommended. One reason for this difference stems from surrounding tissues, such as myometrium and parametrium. These surrounding tissues may tend to show low intensity compared to prostate on DWI, even when b-values are inferior to 2000. In addition, there is a difference in the type of cancer pathology. Prostate cancer consists of adenocarcinoma whereas cervical cancer mainly consists of squamous cell carcinoma, and the ADC value of squamous cell carcinoma tends to be lower than that of adenocarcinoma.^23,24^ This difference may affect the appropriate b-value in each type of cancer.

Our study has a few limitations. First, a precise correlation with pathological specimen was not performed, as some cases were confirmed by biopsy and treated with chemoradiotherapy. However, since the focus of the study was the signal intensity of the tumor, this should not have affected the results. Secondly, small lesions less than 10 mm were excluded, and this may have led to a certain bias in case selection. With the use of high resolution DWI, smaller tumor may also be included in the next step. Thirdly, our study included both 1.5T and 3.0T MR units. It is reported that no significant difference in ADC in upper abdominal organs except for some cases.^25^ However, the increase of SNR at 3T units may have some impact on image quality of computed DWI, and further study is required on this aspect.

In conclusion, computed DWI with b-values of 1300 or 1600 is significantly superior to computed DWI with b-values of 800, 1000 or 2000 in terms of tumor conspicuity and total image quality. Computed DWI with b-values of 1300 or 1600 may thus be recommended for the clinical evaluation of the extent of cervical cancer.

## Figures and Tables

**Table 1.** The results of the scores for tumor conspicuity, signal suppression of the fat in the imaged area, and total image quality

	b-value (s/mm^2^)	800	1000	1300	1600	2000
Tumor conspicuity	Reader 1	2.471	2.965	3.341	3.388	2.988
	Reader 2	2.506	2.988	3.365	3.435	2.882
Signal suppression of the fat in the imaged area	Reader 1	3.082	3.000	2.224	1.906	1.435
	Reader 2	3.012	3.000	2.188	1.765	1.224
Total image quality	Reader 1	2.553	2.953	3.341	3.365	2.824
	Reader 2	2.682	3.000	3.376	3.188	2.318

**Table 2.** Suppression of the signal intensity of the endocervical canal

b-value (s/mm^2^)	800	1000	1300	1600	2000	Not suppressed
Reader 1	0	1	16	7	2	1
Reader 2	0	3	11	10	2	1

**Table 3.** CRs of tumor to myometrium, endocervical canal, endometrium and fat

	b-value (s/mm^2^)	800	1000	1300	1600	2000
tumor / myometrium	average	0.254	0.281	0.319	0.355	0.399
	SD	0.117	0.119	0.127	0.141	0.162
tumor / endocervical canal	average	0.170	0.199	0.242	0.283	0.335
	SD	0.092	0.081	0.082	0.100	0.134
tumor / endometrium	average	0.035	0.077	0.138	0.196	0.265
	SD	0.131	0.133	0.153	0.183	0.225
tumor / fat	average	0.618	0.575	0.504	0.424	0.307
	SD	0.073	0.080	0.094	0.113	0.144

## References

[1] Saslow D Solomon D Lawson HW Myers American Cancer Society, American Society for Colposcopy and Cervical Pathology, and American Society for Clinical Pathology screening guidelines for the prevention and early detection of cervical cancer. Am J Clin Pathol 2012; 137:516–542. 22431528 10.1309/AJCPTGD94EVRSJCG

[2] Rob L Skapa P Robova H. Fertility-sparing surgery in patients with cervical cancer. Lancet Oncol 2011; 12:192–200. 20619737 10.1016/S1470-2045(10)70084-X

[3] Dhanda S Thakur M Kerkar R Jagmohan P. Diffusion-weighted imaging of gynecologic tumors: diagnostic pearls and potential pitfalls. Radiographics 2014; 34:1393–1416. 25208287 10.1148/rg.345130131

[4] Naganawa S Sato C Kumada H Ishigaki T Miura S Takizawa O. Apparent diffusion coefficient in cervical cancer of the uterus: comparison with the normal uterine cervix. Eur Radiol 2005; 15:71–78. 15538578 10.1007/s00330-004-2529-4

[5] Fujii S Matsusue E Kanasaki Y Detection of peritoneal dissemination in gynecological malignancy: evaluation by diffusion-weighted MR imaging. Eur Radiol 2008; 18:18–23. 17701040 10.1007/s00330-007-0732-9

[6] Seo JM Kim CK Choi D Kwan Park B. Endometrial cancer: utility of diffusion-weighted magnetic resonance imaging with background body signal suppression at 3T. J Magn Reson Imaging 2013; 37:1151–1159. 23150461 10.1002/jmri.23900

[7] Beddy P Moyle P Kataoka M Evaluation of depth of myometrial invasion and overall staging in endometrial cancer: comparison of diffusion-weighted and dynamic contrast-enhanced MR imaging. Radiology 2012; 262:530–537. 22114239 10.1148/radiol.11110984

[8] Park JJ Kim CK Park SY Park BK. Parametrial invasion in cervical cancer: fused T_2_-weighted imaging and high-b-value diffusion-weighted imaging with background body signal suppression at 3T. Radiology 2015; 274:734–741. 25299787 10.1148/radiol.14140920

[9] Blackledge MD Leach MO Collins DJ Koh DM. Computed diffusion-weighted MR imaging may improve tumor detection. Radiology 2011; 261:573–581. 21852566 10.1148/radiol.11101919

[10] Ueno Y Takahashi S Kitajima K Computed diffusion-weighted imaging using 3-T magnetic resonance imaging for prostate cancer diagnosis. Eur Radiol 2013; 23:3509–3516. 23881300 10.1007/s00330-013-2958-z

[11] Bihan DL Breton E. Imagerie de diffusion *in vivo* par résonance magnétique nucléaire. C R Acad Sci (Paris) 1985; 301:1109–1112.

[12] Landis JR Koch GG. The measurement of observer agreement for categorical data. Biometrics 1977; 33:159–174. 843571

[13] Maas MC Fütterer JJ Scheenen TW. Quantitative evaluation of computed high B value diffusion-weighted magnetic resonance imaging of the prostate. Invest Radiol 2013; 48:779–786. 23907102 10.1097/RLI.0b013e31829705bb

[14] Takahara T Imai Y Yamashita T Yasuda S Nasu S Van Cauteren M. Diffusion-weighted whole body imaging with background body signal suppression (DWIBS): technical improvement using free breathing, STIR and high resolution 3D display. Radiat Med 2004; 22:275–282. 15468951

[15] Ueno Y Kitajima K Sugimura K Ultra-high b-value diffusion-weighted MRI for the detection of prostate cancer with 3-T MRI. J Magn Reson Imaging 2013; 38: 154–160. 23292979 10.1002/jmri.23953

[16] Rosenkrantz AB Chandarana H Hindman N Computed diffusion-weighted imaging of the prostate at 3T: impact on image quality and tumour detection. Eur Radiol 2013; 23:3170–3177. 23756956 10.1007/s00330-013-2917-8

[17] Ohgiya Y Suyama J Seino N Diagnostic accuracy of ultra-high-b-value 3.0-T diffusion-weighted MR imaging for detection of prostate cancer. Clin Imaging 2012; 36:526–531. 22920357 10.1016/j.clinimag.2011.11.016

[18] Metens T Miranda D Absil J Matos C. What is the optimal b value in diffusion-weighted MR imaging to depict prostate cancer at 3T? Eur Radiol 2012; 22:703–709. 21971824 10.1007/s00330-011-2298-9

[19] Kitajima K Takahashi S Ueno Y Clinical utility of apparent diffusion coefficient values obtained using high b-value when diagnosing prostate cancer using 3 tesla MRI: comparison between ultra-high b-value (2000 s/mm^2^) and standard high b-value (1000 s/mm^2^). J Magn Reson Imaging 2012; 36:198–205. 22371381 10.1002/jmri.23627

[20] Katahira K Takahara T Kwee TC Ultra-high-b-value diffusion-weighted MR imaging for the detection of prostate cancer: evaluation in 201 cases with histopathological correlation. Eur Radiol 2011; 21:188–196. 20640899 10.1007/s00330-010-1883-7

[21] Kim CK Park BK Kim B. High-b-value diffusion-weighted imaging at 3T to detect prostate cancer: comparisons between b values of 1,000 and 2,000 s/mm^2^. Am J Roentgenol 2010; 194:W33–W37. 20028888 10.2214/AJR.09.3004

[22] Grant KB Agarwal HK Shih JH Comparison of calculated and acquired high b value diffusion-weighted imaging in prostate cancer. Abdom Imaging 2015; 40:578–586. 25223523 10.1007/s00261-014-0246-2 PMC5540661

[23] Liu Y Bai R Sun H Liu H Wang D. Diffusion-weighted magnetic resonance imaging of uterine cervical cancer. J Comput Assist Tomogr 2009; 33:858–862. 19940650 10.1097/RCT.0b013e31819e93af

[24] Lin Y Li H Chen Z Correlation of histogram analysis of apparent diffusion coefficient with uterine cervical pathologic finding. Am J Roentgenol 2015; 204:1125–1131. 25905952 10.2214/AJR.14.13350

[25] Rosenkrantz AB Oei M Babb JS Niver BE Taouli B. Diffusion-weighted imaging of the abdomen at 3.0 Tesla: image quality and apparent diffusion coefficient reproducibility compared with 1.5 Tesla. J Magn Reson Imaging 2011; 33:128–135. 21182130 10.1002/jmri.22395

